# Comment on Yeste et al. Polyphenols and IUGR Pregnancies: Intrauterine Growth Restriction and Hydroxytyrosol Affect the Development and Neurotransmitter Profile of the Hippocampus in a Pig Model. *Antioxidants* 2021, *10*, 1505

**DOI:** 10.3390/antiox11050833

**Published:** 2022-04-25

**Authors:** Glòria Garrabou, Ana Sandra Hernández, Mariona Guitart-Mampel, Elena Escalada-Casellas, Gemma Malats-Revelles, Sara Castro-Barquero, Ana María Ruiz-León, Kilian Vellvé, Rosa Casas, Francesc Cardellach, Fàtima Crispi, Francesc Josep García-García

**Affiliations:** 1 Muscle Research and Mitochondrial Function Laboratory, Cellex-IDIBAPS, Faculty of Medicine and Health Sciences, University of Barcelona, 08036 Barcelona, Spain; mguitart@clinic.cat (M.G.-M.); elena.escaladac@gmail.com (E.E.-C.); gmalatre7@gmail.com (G.M.-R.); fcardell@clinic.cat (F.C.); 2 Internal Medicine Unit, Medicine Department, Hospital Clínic of Barcelona, 08036 Barcelona, Spain; sacastro@clinic.cat (S.C.-B.); amruiz@clinic.cat (A.M.R.-L.); rcasas1@clinic.cat (R.C.); 3 Biomedical Network Research Centre on Rare Diseases (CIBERER), Instituto de Salud Carlos III, 28029 Madrid, Spain; ashernandez@clinic.cat (A.S.H.); kvellve@clinic.cat (K.V.); fcrispi@clinic.cat (F.C.); 4 BCNatal—Barcelona Centre for Maternal-Foetal and Neonatal Medicine (Hospital Clínic and Hospital Sant Joan de Déu), IDIBAPS, University of Barcelona, 08036 Barcelona, Spain; 5 Cardiovascular Risk, Nutrition and Aging Laboratory, Cellex-IDIBAPS, Faculty of Medicine and Health Sciences, University of Barcelona, 08036 Barcelona, Spain; 6 Biomedical Network Research Centre on Obesity and Nutrition Physiopathology (CIBEROBN), Instituto de Salud Carlos III, 28029 Madrid, Spain; 7 Mediterranean Diet Foundation, 08021 Barcelona, Spain

**Keywords:** intrauterine growth restriction, hydroxytyrosol, tyrosol, supplementation, balanced diet, Mediterranean diet

## Abstract

Intrauterine growth restriction (IUGR) affects 5–10% of newborns and increases the risks of intrauterine demise, neonatal morbidity, and death. In their recent publication, Yeste et al. found the benefits of hydroxytyrosol supplementation on brain remodeling from an IUGR pig model. Additionally, we found a significant decrease in phenolic alcohol (tyrosol and hydroxytyrosol) intake in IUGR pregnant women. Altogether, these findings support the notion that dietetic interventions, through supplementation but mostly via a balanced diet, can ameliorate IUGR complications. Furthermore, diet intervention combined with early biomarkers may allow clinicians to eventually anticipate IUGR diagnosis and help avoid one of the most frequent causes of newborn mortality and morbidity.

Intrauterine growth restriction (IUGR) is a pathological condition characterized by the limitation of the fetus to grow to its genetically determined size [1]. The World Health Organization estimates that one in seven babies worldwide is born with a low birth weight, and this is a major cause of newborn mortality, with a risk of death of around 2.5% [2,3]. Additionally, long-term effects on adulthood have been proven in IUGR newborns. The unfavorable intrauterine environment present in IUGR creates altered organogenesis, leading to cardiovascular and neurological remodeling and other suboptimal organ developments. Therefore, cardiovascular disease in adulthood has been associated with IUGR in large epidemiological studies through type 2 diabetes, obesity, hypertension, dyslipidemia, or insulin resistance [4]. Moreover, IUGR is associated with an increased risk of neonatal and adult diseases; for instance, abnormal fetal growth increases the risk of cerebral palsy in children and other short- and long-term neurological disorders, such as behavioral disabilities, attention difficulties, and even epilepsy [5].

An article published by Yeste et al. [6] shows that treatment with hydroxytyrosol (HTX) improves the neurological alterations of a previously validated porcine model of IUGR [7,8,9]. The IUGR model used by Yeste et al. was obtained by limiting the amount of food to 50% of daily maintenance requirements. This reduction in nutrient supply triggers “brain-sparing”, an increase in blood flow to the brain to ensure correct neural development. This situation has been observed in pigs but also in humans, and conditions fetus organogenesis during the rest of the pregnancy and further in adult life [10,11].

The final destiny of nutrients and oxygen at the cellular level is the mitochondria. The mitochondria are the powerhouse of the cell and the final subject responsible for intermediary metabolism. Additionally, in pathological conditions, they are also the main source of oxidative stress and cell death by apoptosis [12]. In this sense, and based on our findings, Yeste et al.’s IUGR model is potentially diminishing mitochondrial function and impairing oxidative stress levels. These results would match with previous observations from our group where the mitochondrial function was compromised in IUGR human newborns and placenta [13], as well as in offspring of an IUGR rabbit model [14].

To summarize, different kinds of alterations, from pathophysiological to the molecular level, have been found not only in animal models, such as pigs or rabbits, but also in early and long-term human IUGR subjects. Despite all this evidence accumulated and related to the deleterious effects of IUGR—many of them related to metabolic and bioenergetic imbalance—therapeutic interventions are lacking. One of the reasons underscoring this lack of therapeutic options is the ethical concerns raised by pharmacological treatment during pregnancy and its potential consequences on fetuses’ development or long-term effects on adulthood.

Interestingly, dietetic interventions during pregnancy, based on dietary recommendations and/or supplementation with nutraceutics, open a novel approach to confronting disease during the fetal period. The importance of diet during pregnancy to prevent different pathologies has been extensively demonstrated [15,16]. For instance, folic acid to prevent neural tube defects, iodine to prevent cretinism, and calcium and vitamin D to reduce gestational hypertensive disorders are usually provided during pregnancy. Furthermore, iron supplementation, primarily recommended to reduce anemia during pregnancy, has proven to significantly reduce the risk of restricted growth pathologies by 20% [17].

In their work, Yeste et al. hypothesize that HTX supplementation in the maternal diet could partially or totally reverse the effects of IUGR. Their results in the pig model demonstrated that HXT supplementation reverses the altered immunohistochemical damage of the hippocampus as well as the neurotransmitter profile to values close to normality. In accordance with these results, we collected preliminary data on dietetic habits in a cohort of IUGR pregnant women (*n* = 15) vs. pregnant controls (*n* = 28), and interestingly, there is a relationship between Yeste et al.’s work and our results. In our study, IUGR is defined according to the established clinical criteria [18]. The dietary data were obtained, in the immediate postpartum—with comparable gestational age across all participants—by completing one Food Frequency Questionnaire (FFQ), validated for pregnancy, that included 151 nutritional items [19]. Dietary polyphenol intake was estimated by multiplying the polyphenol content in food (mg/100 g of food) by the daily consumption of each food (g/day), as described elsewhere [20]. The total polyphenol intake and polyphenol subclasses were calculated as the sum of all individual polyphenol intakes from the food sources reported from the FFQ. The obtained results were compared between groups using nonparametric statistics (U Mann–Whitney). In our population, despite no differences being found between the caloric intakes (CTL: 2335 ± 659.7 Kcal/day vs. IUGR: 2025 ± 698.0 Kcal/day), the IUGR mother group showed a significantly lower intake of phenolic alcohols (Tyrosol and HTX) compared to the control group (CTL: 32.02 ± 18.33 vs. IUGR: 19.36 ± 14.97 mg/day) (Figure 1). These data suggest that a lower intake of phenolic alcohols during human pregnancy increases the risk of IUGR outcomes, despite no causality being established.

However, it is important to note that Yeste et al. observed beneficial effects when the supplementation was performed from day 35 of pregnancy onwards. A swine’s gestation time is about 120 days, and in their work, piglets were retrieved on day 100. According to this chronology, supplementation was started at the beginning of the second trimester. Interestingly, several studies have demonstrated the association between adherence to a balanced diet, such as the Mediterranean diet, and a decreased risk of IUGR [21,22]. This is an important aspect since human IUGR is not confirmed until the third trimester. In fact, clinically, IUGR is defined by an estimated fetal growth (EGF) < 3rd percentile adjusted for gestational age (GA) or by the presence of an EFG < 10th percentile for GA associated with a pathological Doppler ultrasound performed in the third trimester with alterations of the cerebral–umbilical flow or the uterine arteries [18].

Consequently, the diagnosis arrives late, preventing potential benefits from HXT or any other type of intervention. In this sense, it is important to develop accurate early biomarkers in parallel with effective treatments to facilitate the intervention and benefit of positive dietary effects.

Diet is the most significant factor for developmental disability and premature death [21]. Interestingly, diet can also protect against several pathologies, such as cardiovascular disease or cognitive decline [23,24]. A balanced diet, such as the Mediterranean diet (rich in fish, olive oil, fruits, vegetables, whole grains, and legumes/nuts), has a protective role on maternal and newborn health during pregnancy [25,26]. In fact, recent publications demonstrate the ability of combinations of nutrients [27] and, specifically, the Mediterranean diet, to reduce adverse perinatal outcomes [28]. The benefits of the diet are, in part, because this diet is rich in mono- and polyunsaturated fatty acids and antioxidant polyphenols, including TYR and HXT, and many other active/protective molecules supplied by components such as virgin olive oil. Additionally, many of the foods included in the Mediterranean diet, such as fruits, vegetables, and nuts, are a great source of antioxidant polyphenols [29]. The present data support this notion in affected patients (IUGR pregnancies) and may be helpful to prevent this obstetric complication and deepen the mechanisms behind its beneficial effects. However, there is a crucial need to find early biomarkers for IUGR diagnosis in the first trimester of pregnancy, when therapeutic interventions are still affordable to prevent or minimize IUGR complications.

Interestingly, individual dietary patterns based on personalized medicine will probably set the path for novel preventive and therapeutic strategies in the near future.

## Figures and Tables

**Figure 1:**
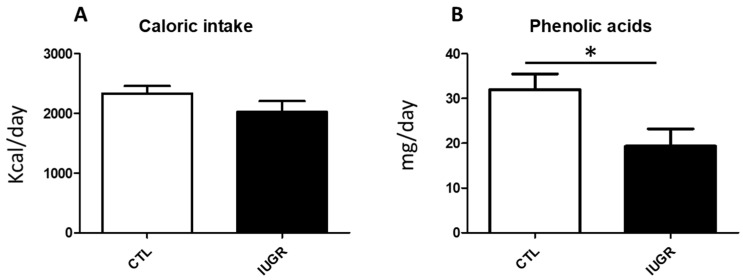
Caloric and phenolic alcohol (Tyrosol and Hydroxytyrosol) daily intake in control (CTL) vs. intrauterine growth restriction (IUGR) in pregnant women. (**A**). Conserved trend of caloric intake between CTL and IUGR pregnant women. (**B**). The daily intake of phenolic alcohols (tyrosol and HXT) is significative lower in IUGR when compared to CTL pregnant women. Results are expressed as means and standard error of the mean (SEM) (* *p* < 0.05).

## Data Availability

The data presented in this study are available on request from the corresponding author.
